# Associations Between Stress, Psychological Capital, Well‐Being, Job Burnout, and Turnover Intention Among Chinese Nurses: A National Cross‐Sectional Study

**DOI:** 10.1155/jonm/1100208

**Published:** 2026-06-30

**Authors:** Di Liu, Chunxiao Long, Yuxuan Du, Zihan Hua, Zhihao Jia, Mengxiong Wu, Lei Shi

**Affiliations:** ^1^ School of Marxism, Harbin Medical University, Harbin, China, hrbmu.edu.cn; ^2^ School of Health Management, Guangzhou Medical University, Guangzhou, China, gzhmc.edu.cn; ^3^ School of Health Management, Harbin Medical University, Harbin, China, hrbmu.edu.cn; ^4^ Health Publicity and Education Center of Guangdong Province, Guangzhou, China; ^5^ School of Health Management, Southern Medical University, Guangzhou, China, fimmu.com; ^6^ Philosophy and Social Sciences Key Laboratory of Guangdong Higher Education Institutes for Health Governance Based on Big Data Utilization, Guangzhou Medical University, Guangzhou, China, gzhmc.edu.cn; ^7^ Key Laboratory of Philosophy and Social Sciences of Colleges and Universities in Guangdong Province for Collaborative Innovation of Health Management Policy and Precision Health Service, Southern Medical University, Guangzhou, China, fimmu.com

**Keywords:** burnout, nurses, psychological capital, stress, turnover intention, well-being

## Abstract

**Background:**

The shortage of nurses is a worldwide issue. Turnover intention among nurses plays a critical role in shaping workforce stability and healthcare service quality. The inter‐relationships between stress, psychological capital, job burnout, turnover intention, and well‐being in nurses have not been fully clarified and warrant further study.

**Aim:**

This study aimed to investigate how stress affects turnover intention in Chinese nurses, focusing on the serial mediation of psychological capital and job burnout and the moderation of well‐being.

**Methods:**

Using a cross‐sectional study design, registered nurses were recruited from 21 hospitals in eastern, central, and western China, with 4865 valid responses collected. Stress, psychological capital, well‐being, job burnout, and turnover intention were measured using validated instruments. Associations among the variables were examined using a moderated serial mediation model (PROCESS Model 91), adjusting for demographic and occupational covariates.

**Results:**

Stress was positively associated with turnover intention (*r* = 0.380, *p* < 0.01) and job burnout (*r* = 0.568, *p* < 0.01) but negatively associated with psychological capital (*r* = −0.374, *p* < 0.01) and well‐being (*r* = −0.477, *p* < 0.01). Stress had a significant total effect on turnover intention (*c* = 0.290, *p* < 0.001), of which the direct effect (c’ = 0.142, *p* < 0.001) accounted for 48.97% of the total effect. Psychological capital and job burnout significantly mediated this relationship. Specifically, the indirect effect through psychological capital was 0.016, accounting for 5.51% of the total effect, whereas the indirect effect through job burnout was 0.091, accounting for 31.38% of the total effect. Stress affected turnover intention through the serial mediation of psychological capital and job burnout (indirect effect = 0.041, 14.14% of the total effect). Well‐being significantly moderated the association between psychological capital and job burnout, with an index of moderated mediation of −0.0008. The serial indirect effect decreased as well‐being increased.

**Conclusion:**

Psychological capital and job burnout represent important mechanisms linking stress to nurses’ turnover intention, and well‐being moderates the pathway between psychological capital and job burnout. These findings provide evidence for multidimensional interventions to reduce nurses’ turnover intention, including strengthening psychological capital assessment, improving occupational well‐being, and optimizing workplace support systems.

**Implications for Nursing Management:**

Nurse administrators should adopt comprehensive strategies addressing stress, psychological capital, well‐being, and job burnout to lower nurses’ turnover intention and maintain workforce stability.

## 1. Introduction

Nurse shortages represent a global challenge [1]. The World Health Organization’s State of the World’s Nursing Report 2025 indicates that the global nursing workforce gap reached approximately 5.9 million in 2023, and total shortages in the global health workforce are expected to surpass 11 million by 2030 [2]. In China, the nursing workforce is also under considerable pressure. Despite a steady increase in the number of registered nurses, the country still faces persistent workforce strain due to its large population base, rising demand for medical services, and accelerated population aging. By the end of 2023, China had 5.63 million registered nurses, with approximately 4 nurses per 1000 population, while adults aged 60 years and above accounted for 21.1% of the total population, creating additional demand for nursing care in both hospitals [3, 4]. Against this background, Chinese nurses are confronted not only with heavy clinical workloads but also with increasing demands for high‐quality, continuous, and complex care, which further intensify workforce pressure.

Turnover intention is defined as the perceived probability that an employee will voluntarily leave their position in the near future and is considered a key indicator of job retention intention and a predictor of actual turnover among healthcare workers [5, 6]. Previous studies have shown that turnover intention may translate into actual turnover behavior, which can undermine the continuity and quality of healthcare services [7]. At the same time, the intensification of global population aging has expanded the rigid demand for nursing services, and the essential role of nurses in healthcare provision has made the nurse shortage an even more pressing issue. Consequently, research has increasingly focused on the factors influencing turnover intention [8]. Exploring the underlying mechanisms and influencing factors of nurses’ turnover intentions is crucial for developing targeted interventions aimed at alleviating the nursing shortage, and holds significant theoretical and practical implications [9].

Nurse stress refers to a state of subjective tension and maladaptation experienced by nurses due to high risk, heavy workload, complex interpersonal relationships, and poor working conditions in nursing practice [10]. Studies have reported that 89.87% of nurses perceive their stress levels as moderate to high [11]. Following the outbreak of the COVID‐19 pandemic, nurses have become more vulnerable to chronic stressors such as excessive workloads, night‐shift rotations, and insufficient human resources [12, 13]. As a profession marked by high demands and intense pressure, nursing involves heavy workloads, diverse and unpredictable patient needs, and highly stressful care environments, which may increase stress levels and lead to job burnout and turnover intention. Prior evidence indicates that elevated stress is likely to induce job burnout, and job burnout subsequently heightens the risk of turnover among nurses [14, 15]. Moreover, evidence suggests that nurses’ psychological capital plays a partial mediating role between stress and job burnout, with higher psychological capital buffering the adverse effects of stress on burnout [16].

Psychological capital is defined as a positive psychological state manifested during individual growth and development, encompassing four core dimensions: self‐efficacy, resilience, hope, and optimism [17, 18]. In recent years, psychological capital has gained growing scholarly attention in occupational contexts, especially within nursing research [19]. According to the conservation of resources theory [20], individuals with richer psychological resources are better able to cope with work stress and maintain adaptive functioning in demanding environments. Specifically, nurses with higher self‐efficacy may be more inclined to initiate resource acquisition strategies, while resilience is more closely associated with resource defense and recovery mechanisms, helping individuals restore adaptive functioning after resource depletion. Hope and optimism may promote sustained resource acquisition and resource gains through pathway investment and positive expectations, respectively, thereby forming distinct yet complementary resource conservation pathways in nursing practice. Evidence indicates that nurses’ psychological capital is significantly associated with alleviation of job burnout and exerts a beneficial effect on reducing turnover intention [18, 21]. Studies conducted by Luthans et al. have further shown that employees with higher levels of psychological capital exhibit lower turnover intention and make greater contributions to organizational performance [22]. Recent research has further highlighted the role of psychological capital in the nursing field. A study of newly qualified nurses found that psychological capital significantly mediated the relationship between professional identity and turnover intention [8]. Furthermore, other studies have found that nurses with higher levels of psychological capital are better equipped to cope with workplace challenges and experience greater job satisfaction [23]. Additional research has confirmed from another perspective that nurses with lower levels of psychological capital are more prone to emotional exhaustion and fatigue, thereby exacerbating symptoms of job burnout [24].

Job burnout refers to a state of physical and mental fatigue and exhaustion resulting from prolonged exposure to stress [25, 26]. This syndrome is now prevalent in the nursing profession, with evidence suggesting that as many as 50% of nurses experience job burnout, resulting in substantial negative personal consequences, impaired work functioning, and potential threats to patient safety [27]. On the one hand, job burnout is influenced by a variety of factors. Research has found that emotional labor among young nurses or hospital administrative staff indirectly affects their job burnout through social support and emotional regulation [28, 29], while other studies have indicated that an increase in negative emotions and a decrease in job satisfaction lead to higher levels of burnout [30]. On the other hand, job burnout can also lead to adverse consequences, and multiple studies on the antecedents of turnover intention have shown that job burnout positively predicts turnover intention [17, 31].

Well‐being is a key component of nurses’ mental health, typically encompassing positive emotions or experiences of joy, reduced negative emotions, and an individual’s overall satisfaction with life at a given point in time [25]. Previous research suggests that well‐being may further improve behavior and work performance by enhancing an individual’s positive psychological capital [23]. Furthermore, studies have found a negative association between well‐being and job burnout [32, 33]. However, the specific mechanisms through which well‐being influences turnover intention require further investigation [34].

Against the backdrop of deepening healthcare system reforms and accelerating population aging, the nursing profession is facing unprecedented challenges. Investigating the complex relationships among stress, psychological capital, job burnout, turnover intention, and well‐being among nurses is of substantial practical significance for improving nursing human resource management, optimizing professional support systems, and promoting nurses’ well‐being and the sustainable development of the profession. Therefore, this study aimed to examine the direct effects of stress on job burnout and turnover intention, to investigate the mediating and serial mediating roles of psychological capital and job burnout in the relationship between stress and turnover intention, and to explore whether well‐being plays a moderating role in these associations. Grounded in conservation of resources theory, this study posits that, in the nursing work context, stress can be regarded as an important job demand that continuously consumes nurses’ emotional and psychological resources. Psychological capital, as a positive personal psychological resource, helps nurses better cope with work challenges, buffer negative influences, and maintain a positive occupational state. When stress persists and psychological capital is insufficient, nurses are more likely to experience job burnout, such as emotional exhaustion and reduced sense of accomplishment. As stress accumulates, psychological capital gradually declines, and job burnout intensifies, nurses’ turnover intention may also increase accordingly. Meanwhile, well‐being, as a positive psychological state, may play a protective and buffering role in the process of stress and resource depletion, thereby alleviating adverse effects. Accordingly, a moderated serial mediation model was adopted to examine the relationships among stress, psychological capital, well‐being, job burnout, and turnover intention. Based on this framework, the following hypotheses were proposed:

 H1: Psychological capital mediates the relationship between stress and turnover intention.
 H2: Job burnout mediates the relationship between stress and turnover intention.
 H3: Psychological capital and job burnout jointly play a serial mediating role in the relationship between stress and turnover intention.
 H4: Well‐being moderates the effect of psychological capital on job burnout and further moderates the serial mediating effect of stress on turnover intention through psychological capital and job burnout.

## 2. Materials and Methods

### 2.1. Design and Sample

This study was reported in accordance with the Strengthening the Reporting of Observational Studies in Epidemiology (STROBE) statement. The STROBE checklist for cross‐sectional studies was used to ensure transparent reporting of the study design, data collection, statistical analysis, and interpretation of findings.

This study employed purposive sampling between March and July 2021, selecting one province from each of three regions in China: eastern (Zhejiang), central (Heilongjiang), and western (Chongqing). Within each province, two hospitals were randomly selected from prefecture‐level cities with the highest GDP, three from cities with medium GDP, and two from cities with the lowest GDP, and healthcare staff in these hospitals were surveyed using questionnaires. The inclusion criteria were: (1) registered nurses; (2) at least 1 year of hospital work experience; (3) provision of informed consent; and (4) voluntary participation in the survey, while nursing students working in hospitals were excluded.

### 2.2. Data Collection

Before the formal survey and data collection, all investigators received standardized training. Meanwhile, 100 nurses were selected from each region for a pilot survey, and based on the pilot results and expert feedback, the scale items were revised and the final questionnaire was developed, which was distributed to nurses after coordination with hospital administrators and approval from department heads. Before the formal investigation, researchers explained the purpose and content of the survey in detail, informed participants of their right to participate voluntarily or refuse to answer any question, and assured them that the survey was confidential and that all collected data would be used solely for scientific research and handled under strict confidentiality.

The survey was conducted after nurses provided voluntary consent and signed informed consent forms, and 250 nurses were selected from each hospital using a random number table method; a total of 5250 questionnaires were distributed, of which 4865 were valid after excluding questionnaires with obvious errors, incorrect responses to lie‐detection items, or incomplete answers, yielding a valid response rate of 92.67% [35].

To ensure sample size adequacy, a priori sample size estimation was performed for multiple regression analysis based on Cohen’s *f* ^2^, where *f* ^2^ = *R* ^2^/(1‐*R* ^2^). Assuming a small effect size (*f* ^2^ = 0.02), an alpha level of 0.05, a power of 0.90, and 20 predictors, the minimum required sample size was estimated to be 1323. A post hoc power analysis further showed that, with the final sample size of 4865, the achieved statistical power was greater than 0.99, indicating that the study had sufficient power for the planned analyses.

### 2.3. Measures

The questionnaire comprised six components, including sociodemographic characteristics, stress, psychological capital, well‐being, job burnout, and turnover intention scales.

#### 2.3.1. Sociodemographic Variables

Sociodemographic data were collected using a structured questionnaire, including gender, age, marital status, child status, department, years of nursing experience, educational attainment, monthly income (CNY), and average daily working hours during the past month (hours).

#### 2.3.2. Stress

The Depression Anxiety Stress Scales (DASS‐42), developed by Lovibond in 1995, were designed to evaluate levels of depression, anxiety, and stress in adults [36]. The scale was later revised by Antony into the abbreviated DASS‐21, consisting of 21 items [37]. Due to its brevity, practicality, and rapid administration, the scale has been translated into numerous languages and applied extensively in international research. Responses are rated on a four‐point Likert scale ranging from 0 (does not apply) to 3 (applies always), with higher scores indicating more severe levels of depression, anxiety, and stress. In the present study, nurses’ stress was assessed using the stress subscale of the DASS. In this study, the stress subscale demonstrated excellent internal consistency, with a Cronbach’s *α* of 0.94, and showed good construct validity, as indicated by a KMO value of 0.906.

#### 2.3.3. Psychological Capital

Psychological capital was assessed with the Chinese version of the Nurse Psychological Capital Questionnaire (PCQ‐R for Chinese), which was originally developed by Luthans and colleagues and later adapted by Chinese researchers [16, 38]. This scale was developed in accordance with the nursing context in China and consists of 24 items covering four dimensions: self‐efficacy, hope, resilience, and optimism, and has been widely used in previous studies. Each item is rated on a six‐point Likert scale from 1 to 6 (1 = strongly disagree; 6 = strongly agree), with higher scores indicating higher levels of psychological capital. In this study, the overall scale showed excellent internal reliability, with a Cronbach’s *α* of 0.92. Additionally, the KMO value was 0.963.

#### 2.3.4. Job Burnout

Job burnout among nurses was measured using the 22‐item Maslach Burnout Inventory (MBI), which includes three dimensions: emotional exhaustion, depersonalization, and personal accomplishment [35, 39, 40]. Responses were scored using a seven‐point Likert scale ranging from 0 to 6(0 = never; 6 = every day). Items 4, 7, 9, 12, 17, 18, 19, and 21 were reverse‐scored before calculating the total score, with higher total scores indicating higher levels of job burnout. In the present study, the MBI showed excellent internal reliability, with a Cronbach’s *α* of 0.932 and the KMO value was 0.923.

#### 2.3.5. Well‐Being

The World Health Organization Well‐Being Index (WHO‐5) was employed to evaluate nurses’ well‐being during the previous 2 weeks, and prior research has confirmed its reliability, as indicated by Cronbach’s *α*, in Chinese populations [41]. Items are scored from 0 (never) to 5 (always), with higher scores reflecting higher levels of perceived well‐being. Total scores range from 0 to 25, and scores lower than 13 are indicative of poor well‐being. In the present study, the WHO‐5 showed satisfactory internal reliability, with a Cronbach’s *α* of 0.873. Additionally, the KMO value was 0.895.

#### 2.3.6. Turnover Intention

Turnover intention was assessed using the scale developed by Michaels and Spector [42]. This six‐item scale comprises three dimensions: employees’ willingness to leave their current job, their perceived ability to leave, and their perceived availability of external job opportunities. Each item is rated on a four‐point scale reflecting participants’ intentions (1 = never, 2 = rarely, 3 = occasionally, and 4 = often). Total scores range from 6 to 24, with higher scores indicating stronger turnover intention, and the scale has been widely used in surveys of Chinese employees [22]. In the present study, the turnover intention scale demonstrated good internal consistency, with a Cronbach’s *α* of 0.832. Additionally, the KMO value was 0.897.

### 2.4. Data Analysis

All data were analyzed and processed using IBM SPSS Statistics Version 26. First, descriptive analyses were performed to summarize the general characteristics of the study population. Second, because the data did not follow a normal distribution, Spearman correlation analysis was conducted to examine the relationships among stress, psychological capital, job burnout, well‐being, and turnover intention. Finally, a moderated serial mediation model was tested using the SPSS PROCESS macro (Model 91) to determine whether psychological capital and job burnout exerted a serial mediating effect between stress and turnover intention and whether well‐being played a moderating role [43]. Model 91, developed by Hayes, is an effective framework for testing serial mediation effects and has been widely used in previous studies. Based on the proposed hypotheses, this model was applied to examine the serial mediating effects of psychological capital and job burnout on the relationship between stress and turnover intention, as well as the moderating effect of well‐being, with 95% bootstrap confidence intervals, 5000 bootstrap samples, and a significance level of *p* < 0.05. In the conditional effects analysis, lower and higher levels of well‐being were defined as one standard deviation below and above the mean score, respectively. Considering that relevant covariates (gender, marital status, child status, years of nursing experience, educational level, professional title, and employment status) may influence turnover intention, these variables were adjusted for in the model [44, 45].

### 2.5. Ethical Considerations

This study was approved by the institutional ethics committee (approval number: 202132), and all participants were fully informed about the study and participated voluntarily; individuals who were unable to correctly understand or complete the questionnaire, those with mental or psychological disorders, part‐time nurses, nursing interns, or nurses on leave during the survey period were excluded.

## 3. Results

### 3.1. Common Method Bias

This study employed Harman’s single‐factor test, a commonly used approach for evaluating common method bias [46]. The results showed that seven factors had eigenvalues greater than 1, and the first factor accounted for 39.26% of the total variance, which was below the 40% criterion commonly adopted in prior studies, indicating that common method bias was not serious in this study [46].

### 3.2. Sociodemographic Characteristics of Participants

The majority of participants in this study were female (*n* = 4738, 97.4%), and most were aged between 26 and 45 years (*n* = 3503, 72%). Most nurses were unmarried (*n* = 3476, 71.4%), and 1467 nurses (30.2%) had 6–10 years of nursing experience. See Table 1.

**TABLE 1:** Sociodemographic characteristics of participants (*N* = 4865).

Variables		*n*	Percentage (%)
Gender	Female	4738	97.4
	Male	127	2.6

Age (years old)	≤ 25	629	12.9
	26–35	2371	48.7
	36–45	1132	23.3
	46–55	707	14.5
	≥ 56	26	0.6

Marital status	Unmarried	3476	71.4
	Married	1223	25.2
	Divorced/widowed	166	3.4

Child status	Yes	3168	65.1
	No	1697	34.9

Department	Internal medicine	1254	25.8
	Surgery	1218	25.0
	Emergency treatment	166	3.4
	Gynecology	193	4.0
	Medical technology department	112	2.3
	Pediatrics	358	7.4
	ICU	302	6.2
	Operation room	265	5.4
	Others	997	20.5

Years of nursing work	≤ 5	1002	20.6
	6–10	1467	30.2
	11–20	1237	25.4
	21–30	822	16.9
	≥ 31	337	6.9

Educational level	Technical secondary school and below	222	4.6
	Junior college	1199	24.6
	Undergraduate	3353	68.9
	Graduate and above	91	1.9

Monthly income (yuan)	≤ 3000	694	14.3
	3001–5000	2038	41.9
	5001–7000	1265	26.0
	7001–9000	465	9.6
	≥ 9001	403	8.2

The usual daily working hours in the past month (hours)	≤ 6	124	2.5
	7–8	2929	60.2
	9–10	1276	26.2
	11–12	303	6.3
	≥ 13	233	4.8

Abbreviation: ICU = intensive care unit.

### 3.3. Correlations Among Study Variables

Correlation analysis showed that stress was positively correlated with turnover intention (*r* = 0.380, *p* < 0.01) and job burnout (*r* = 0.568, *p* < 0.01), but negatively correlated with psychological capital (*r* = −0.374, *p* < 0.01) and well‐being (*r* = −0.477, *p* < 0.01). Psychological capital was negatively correlated with job burnout (*r* = −0.640, *p* < 0.01) and turnover intention (*r* = −0.338, *p* < 0.01) and positively correlated with well‐being (*r* = 0.502, *p* < 0.01). Job burnout was negatively correlated with well‐being (*r* = −0.588, *p* < 0.01) but positively correlated with turnover intention (*r* = 0.482, *p* < 0.01). Well‐being was negatively correlated with turnover intention (*r* = −0.344, *p* < 0.01) (see Table 2).

**TABLE 2:** Correlations between stress, psychological capital, job burnout, well‐being, and turnover intention.

Variables	*M*	SD	Stress	Psychological capital	Job burnout	Well‐being	Turnover intention
Stress	12.52	5.25	1				
Psychological capital	106.94	15.97	−0.374^∗∗^	1			
Job burnout	52.38	22.41	0.568^∗∗^	−0.640^∗∗^	1		
Well‐being	18.56	6.78	−0.477^∗∗^	0.502^∗∗^	−0.588^∗∗^	1	
Turnover intention	13.24	4.43	0.380^∗∗^	−0.338^∗∗^	0.482^∗∗^	−0.344^∗∗^	1

^∗∗^ *p* < 0.01.

### 3.4. Results of the Moderated Serial Mediation Model

**FIGURE 1:**
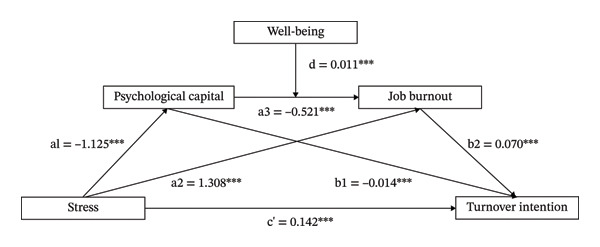
Serial mediation model linking stress, psychological capital, well‐being, job burnout, and turnover intention. *Note:* ^∗∗∗^ *p* < 0.001.

After adjusting for gender, marital status, child status, years of nursing experience, educational attainment, professional title, and employment status, a moderated serial mediation model was specified in which stress served as the independent variable, turnover intention as the dependent variable, psychological capital and job burnout as serial mediators, and well‐being as the moderator (see Figure 1).

The findings indicated that stress had a significant negative effect on psychological capital (a1 = −1.125, 95% CI [−1.202, −1.047]) and a significant positive effect on job burnout (a2 = 1.308, 95% CI [1.220, 1.397]). Psychological capital exerted a negative influence on job burnout (a3 = −0.521, 95% CI [−0.552, −0.491]) and a significant negative influence on turnover intention (*b*1 = −0.014, 95% CI [−0.023, −0.005]), whereas job burnout showed a significant positive effect on turnover intention (*b*2 = 0.070, 95% CI [0.062, 0.077]). In addition, the interaction term between psychological capital and well‐being had a significant positive effect on job burnout (*d* = 0.011, 95% CI [0.007, 0.014]). Stress continued to exert a significant direct effect on turnover intention (c′ = 0.142, 95% CI [0.116, 0.167]). The total effect of stress on turnover intention was 0.290 (see Table 3).

**TABLE 3:** Hypothesized serial mediation model of psychological capital and job burnout between stress and turnover intention (*N* = 4865).

Pathway	Effect	SE	95% CI	*p*	Proportion of total effect (%)
Total effect (*c*): Stress ⟶ Turnover intention	0.290	—	—	—	—
Direct effect (*c* ^′^): Stress ⟶ Turnover intention	0.142	0.013	0.116, 0.167	< 0.001	48.97
a1: Stress ⟶ Psychological capital	−1.125	0.040	−1.202, −1.047	< 0.001	—
a2: Stress ⟶ Job burnout	1.308	0.045	1.220, 1.397	< 0.001	—
a3: Psychological capital ⟶ Job burnout	−0.521	0.015	−0.552, −0.491	< 0.001	—
b1: Psychological capital ⟶ Turnover intention	−0.014	0.005	−0.023, −0.005	0.002	—
b2: Job burnout ⟶ Turnover intention	0.070	0.004	0.062, 0.077	< 0.001	—
d: Psychological capital × Well‐being ⟶ Job burnout	0.011	0.002	0.007, 0.014	< 0.001	—

*Indirect effect*					
Indirect1: Stress ⟶ Psychological capital ⟶ Turnover intention	0.016	0.006	0.005, 0.027		5.51
Indirect2: Stress ⟶ Job burnout ⟶ Turnover intention	0.091	0.006	0.079, 0.103		31.38
Indirect3: Stress ⟶ Psychological capital ⟶ Job burnout ⟶ Turnover intention	0.041	0.003	0.035, 0.047		14.14

*Note:* Path coefficients are regression coefficients. Covariates include gender, marital status, child status, years of nursing experience, educational level, professional title, and employment status.

The mediation analysis showed that psychological capital significantly mediated the relationship between stress and turnover intention, with an effect size of 0.016 (95% CI [0.005, 0.027]), accounting for 5.51% of the total effect. Job burnout likewise significantly mediated the association between stress and turnover intention, yielding an effect size of 0.091 (95% CI [0.079, 0.103]), accounting for 31.38% of the total effect. Moreover, psychological capital and job burnout functioned as serial mediators linking stress to turnover intention, with a significant indirect effect of 0.041 (95% CI [0.035, 0.047]), accounting for 14.14% of the total effect. The direct effect accounted for 48.97% of the total effect. See Table 3.

**FIGURE 2:**
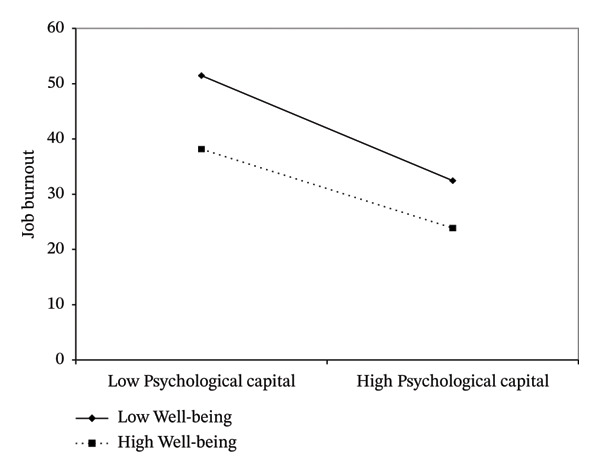
Interaction between psychological capital and well‐being in predicting job burnout. *Note:* Simple slopes are plotted at low (*M* − 1SD) and high (*M* + 1SD) levels of well‐being.

In terms of moderation, the interaction between psychological capital and well‐being significantly predicted job burnout (*B* = 0.011, *p* < 0.001), suggesting that well‐being significantly moderated the association between psychological capital and job burnout (Table 3). Simple slope analyses further indicated (Figure 2) that psychological capital was significantly negatively associated with job burnout at low, mean, and high levels of well‐being. Specifically, when well‐being was low, psychological capital significantly predicted lower job burnout (effect = −0.595, 95% CI [−0.632, −0.559]). At mean levels of well‐being, this negative association remained significant (effect = −0.521, 95% CI [−0.552, −0.491]). At higher levels of well‐being, psychological capital still significantly predicted lower job burnout, although the magnitude of the association was attenuated (effect = −0.448, 95% CI [−0.489, −0.407]). See Table 4.

**TABLE 4:** Conditional effects of psychological capital on job burnout at different levels of well‐being.

Well‐being level	Effect	SE	*t*	*p*	95% CI
Low (*M* − 1SD, *W* = −6.781)	−0.595	0.019	−31.905	< 0.001	0.040, 0.054
Mean (*M*, *W* = 0)	−0.521	0.015	−33.843	< 0.001	0.035, 0.047
High (*M* + 1SD, *W* = 6.781)	−0.448	0.021	−21.542	< 0.001	0.030, 0.041

On this basis, the conditional serial indirect effects of stress on turnover intention via psychological capital and job burnout were further examined. The results showed that at low levels of well‐being, the serial indirect effect was 0.047 (95% CI [0.040, 0.054]). At average levels of well‐being, the conditional indirect effect was 0.041 (95% CI [0.035, 0.047]), and at high levels of well‐being, the conditional indirect effect was 0.035 (95% CI [0.030, 0.041]). All confidence intervals excluded zero, indicating that the conditional serial indirect effects were statistically significant at low, average, and high levels of well‐being. Moreover, the magnitude of the indirect effect decreased as well‐being increased (see Table 5).

**TABLE 5:** Conditional serial indirect effect of stress on turnover intention via psychological capital and job burnout at different levels of well‐being.

Well‐being level	Effect	SE	95% CI
Low (*M* − 1SD, *W* = −6.781)	0.047	0.004	0.040, 0.054
Mean (*M*, *W* = 0)	0.041	0.003	0.035, 0.047
High (*M* + 1SD, *W* = 6.781)	0.035	0.003	0.030, 0.041

The index of moderated mediation was −0.0008 (SE = 0.0002; 95% CI [−0.0012, −0.0005]), further indicating that well‐being significantly moderated the serial mediation pathway.

## 4. Discussion

This study examined whether psychological capital and job burnout mediated the relationship between stress and turnover intention among Chinese nurses, and the results showed that stress had a significant effect on turnover intention, job burnout partially mediated the association between stress and turnover intention, psychological capital and job burnout jointly exerted a mediating effect, and well‐being moderated the relationship between psychological capital and job burnout.

The findings demonstrated that stress was positively correlated with turnover intention among Chinese nurses, in line with prior Chinese research [34, 47], and also similar to international research findings, indicating that this mechanism has cross‐cultural commonality [48]. In the context of globalization and rapid advances in medical technology, healthcare institutions’ heavy reliance on human capital, increasing workloads, and increasingly strained patient–provider relationships have continuously intensified stress among healthcare workers. Particularly during major public health emergencies, nurses experience substantially higher levels of stress [49]. Previous studies have shown that nurses’ turnover intention was significantly higher during the pandemic than before the outbreak [50]. The pandemic exposed nurses to greater stress, which in turn triggered negative emotions such as anxiety and fear. In addition, organizational factors, such as low job control or high job demands, may increase nurses’ psychological burden and further exacerbate turnover intention [51]. Furthermore, Chinese nurses face greater pressure due to workforce shortages, frequent night and rotating shifts, strained nurse–patient relationships, and performance evaluations [52–54]. So, hospitals should focus more on nurses′ mental health, especially the negative impact of public health emergencies, by providing timely support and creating a harmonious work environment to reduce turnover intention. At the government level, efforts should be made to improve the nursing workforce, protect nurses’ rights, promote respect for medical professionals, and enhance incentive systems to reduce stress and attrition, creating a positive cycle.

The results further indicated that nurses’ stress influences turnover intention through psychological capital, such that increased stress undermines psychological capital and subsequently intensifies turnover intention. Prolonged stress not only affects physical health but also impairs mental health, triggering negative emotions [55, 56]. Specifically, prolonged exposure to high‐stress conditions makes nurses more susceptible to fatigue and feelings of powerlessness, gradually depleting key elements of psychological capital, including self‐efficacy, hope, optimism, and resilience, and thereby strengthening turnover intention. Both domestic and international studies have shown that psychological capital is an important protective factor in adapting to stress [57, 58]. In China, nurses are often in high‐demand, emotionally demanding work environments, and they rely more on psychological capital to maintain positive coping strategies [59]. This study emphasizes that enhancing psychological capital should be a core component of strategies to reduce nurses′ turnover intention. Psychological capital is not fixed and can be strengthened through organizational environment. Therefore, at the hospital level, positive feedback mechanisms and a supportive organizational environment can be established. At the government level, nurses′ mental health should be included as an indicator in hospital evaluations, and support for psychological training for staff should be increased.

Nurses’ stress is not only a core predictor of turnover intention but can also operate through a serial mediating pathway involving psychological capital and job burnout. Specifically, stress negatively affects psychological capital, which increases job burnout and ultimately raises turnover intention. This serial mediation pathway accounts for 48.97% of the total effect of stress on turnover intention, showing the important role of psychological capital and job burnout. Previous studies have found that during the pandemic, each unit increase in stress led to a corresponding increase in job burnout [60]. Some studies also suggest that nurses’ job burnout is a common result of long‐term pressure, not just the pandemic [61]. Our findings align with Maslach’s theory, which argues that prolonged exposure to high pressure, combined with insufficient resources, leads to psychological depletion and triggers turnover behavior [41, 60]. Additionally, psychological capital, as a protective resource, can buffer stress and prevent job burnout [18, 21, 34, 62]. Moreover, the COVID‐19 pandemic provided a unique high‐stress context for testing the applicability of conservation of resources theory. Even under conditions of intensified workload and widespread resource depletion, psychological capital remained a protective personal resource that buffered the negative effects of stress on job burnout, thereby supporting the explanatory value of conservation of resources theory in nursing practice. Therefore, understanding the relationship between stress, psychological capital, and job burnout, and how psychological capital helps reduce burnout, can inform strategies to reduce turnover intention. At the hospital level, regular attention should be given to nurses′ work conditions, with timely identification and resolution of any difficulties they encounter. Additionally, work tasks should be reasonably allocated to ensure adequate rest time and a manageable workload. At the governmental level, targeted policies should be developed to strengthen compensation mechanisms and payment standards for shift work and night shifts.

In addition, the study revealed that well‐being can exert a moderating effect on the relationship between psychological capital and job burnout, thereby influencing turnover intention. Specifically, in nursing work with high workload and emotional labor, the higher the well‐being, the more nurses are able to transform psychological capital into coping resources, making them less likely to enter a state of burnout. Well‐being represents a form of positive energy experienced by employees at work and reflects their occupational perceptions derived from the fulfillment of personal values and emotional needs. Previous studies have shown that well‐being enhances psychological capital [47, 63]. Nurses with higher levels of well‐being are more likely to demonstrate proactive behaviors, deliver high‐quality care, and enhance their psychological capital [63]. Moreover, strong well‐being helps maintain positive emotions at work, alleviates job burnout, and consequently reduces turnover intention [64].

Although a growing body of research has focused on the associations among stress, turnover intention, psychological capital, job burnout, and well‐being, studies examining the underlying mechanisms linking these variables remain relatively limited, which constrains the practical applicability of existing findings to some extent. This study provides evidence that psychological capital and job burnout constitute a serial mediation pathway between stress and turnover intention, offering clearer insight into the underlying mechanisms connecting these constructs. In light of these findings, we should implement multidimensional interventions. Hospitals should recognize nurses′ contributions, help them set clear career goals, optimize shift schedules, and improve assessment systems. The government should strengthen policy support to improve nurses′ welfare, salaries, and vacation benefits, while healthcare institutions should provide psychological counseling services to improve job stability and satisfaction. Additionally, differentiated strategies can be adopted based on nurses’ well‐being levels. Given that well‐being moderated the pathway from psychological capital to job burnout, interventions should focus not only on enhancing psychological capital but also on helping nurses transform psychological capital into effective coping resources. For nurses with lower well‐being, regular mental health screening, emotional support, and individualized counseling may help them mobilize psychological capital and reduce burnout. For nurses with higher well‐being and higher psychological capital, managers should also monitor workloads, encourage employees to maintain a work–life balance, and prevent nurses from becoming overburdened. These strategies may be more effective in alleviating job burnout and reducing turnover intention.

Nevertheless, several notable limitations of this study should be acknowledged. First, because a cross‐sectional design was used, causal relationships among variables cannot be established, and longitudinal studies are needed to further verify the identified associations. Second, the data were collected through self‐report measures, which may introduce reporting bias. Finally, data for this study were collected during the COVID‐19 pandemic. Exceptional public health events can make nurses’ work more demanding and stressful [65, 66], potentially leading to increased levels of stress, job burnout, and turnover intention, whilst also affecting psychological capital and well‐being; the strength of the associations between these variables may also be amplified. Despite these limitations, this study still provides valuable insights into the relationship between stress and turnover intention among Chinese nurses.

## 5. Conclusion

In the relationship between stress and turnover intention, psychological capital and job burnout function as serial mediators, while well‐being plays a moderating role between psychological capital and job burnout. The findings of this study provide a structured intervention framework for effectively reducing nurses’ turnover intention, which may serve as a reference for hospital managers and policymakers. It is recommended that policymakers incorporate psychological capital assessment into nurses’ occupational health management systems and enhance well‐being through improvements in salary incentive systems, welfare protection, and the work environment. These measures may help mitigate the negative effects of stress, reduce turnover intention, stabilize the nursing workforce, and promote the sustainable development of healthcare system.

## 6. Implications for Nursing Management

This study provides valuable insights for nursing managers to improve nursing management practices. The findings indicate that while nursing managers should attend to the direct effects of stress on turnover intention, they should also give due consideration to the mediating roles of psychological capital and job burnout, as well as the moderating role of well‐being. First, workforce allocation and scheduling systems should be optimized at the organizational level to alleviate high‐intensity workloads and reduce stress. Second, psychological capital should be enhanced by increasing training opportunities and providing career development support for nurses. Third, early screening and intervention mechanisms for job burnout should be established, including job rotation, flexible scheduling, and psychological support services. Fourth, nurses’ overall well‐being should be enhanced through strengthening organizational culture and improving welfare benefits, thereby amplifying the buffering effect of psychological capital on job burnout. Implementing multidimensional, integrated interventions to reduce nurses’ turnover intention and stabilize the nursing workforce is of significant importance for the field of nursing management.

## Author Contributions

Di Liu and Yuxuan Du conducted literature searches, determined the research theme, and designed the research. Zihan Hua and Zhihao Jia collected the data. Di Liu and Yuxuan Du analyzed the data. Di Liu and Chunxiao Long wrote the manuscript and revised the article. Mengxiong Wu and Lei Shi critically revised the manuscript.

Di Liu, Chunxiao Long, and Yuxuan Du contributed equally to this work.

## Funding

This research was supported by the Heilongjiang Province Philosophy and Social Science Research Planning Project (22GLE375), the Key Research and Development Program Project of Heilongjiang Province (2024ZX12C04), Youth Science and Technology Talent Development Program of Guangdong Association for Science and Technology (SKXRC2025206), Guangdong Basic and Applied Basic Research Foundation (2023A1515010902), and Chronic Disease Management Research Project of the National Health Commission Capacity Building and Continuing Education Center (GWJJMB20210010060)..

## Disclosure

The authors declare that the funding sources had no role in the design, conduct, or reporting of this study. All authors approved the final version for submission.

## Ethics Statement

All study procedures were approved by the Ethics Committee of the University (Approval No. 202132). All participants were fully informed about the purpose of the study and provided written informed consent prior to participation.

## Conflicts of Interest

The authors declare no conflicts of interest.

## Data Availability

The data that support the findings of this study are available upon request from the corresponding author. The data are not publicly available due to privacy or ethical restrictions.
